# Supplementary material for: Wisteria floribunda agglutinin enhances Zaire ebolavirus entry through interactions at specific N-linked glycosylation sites on the virus glycoprotein complex
Source: J Gen Virol. 2025 Jun 6;106(6):002120. doi: 10.1099/jgv.0.002120 (PMC12210185; doi:10.1099/jgv.0.002120)
Supplement: Uncited Supplementary Material 1. [file jgv-106-02120-s001.pdf]

Supplementary Table 1. Positions of *N*-linked glycan sites in the EBOV GP.

	Variant									
Glycan	B1	M1	M2	M3	B12	B13	B14	B16	ΔMLD	Location	Cross-species
N1(40)	+	+	+	+	+	+	+	+	+	Base	Y
N2(204)	+	+	+	+	-	+	+	+	+		Y
N3(228)	+	+	+	+	+	-	-	-	+	Cap	N
N4(238)	+	-	+	+	+	+	+	+	+	Cap	Y
N5(257)	+	+	+	+	+	+	+	+	+	Cap	Y
N6(268)	+	+	-	+	+	+	+	+	+	Cap	Y
N7(296)	+	+	+	-	+	+	+	+	+	Cap	N
N8(317)	+	+	+	+	+	+	+	+	-	MLD	Y
N9(333)	+	+	+	+	+	+	+	+	-	MLD	Y
N10(346)	+	+	+	+	+	+	+	+	-	MLD	N
N11(386)	+	+	+	+	+	+	+	+	-	MLD	N
N12(413)	+	+	+	+	+	+	+	+	-	MLD	Y
N13(436)	+	+	+	+	+	+	+	+	-	MLD	Y
N14(454)	+	+	+	+	+	+	+	+	-	MLD	N
N15(462)	+	+	+	+	+	+	+	+	-	MLD	Y
N16(563)	+	+	+	+	+	+	+	+	+	GP2	Y
N17(618)	+	+	+	+	+	+	+	+	+	GP2	Y

Supplementary Table 2. IC_{50} values of KZ52 against EBOV-C15 and EBOV-C15-ΔMLD in the presence and absence of WFA lectin at a concentration of 10 µg.mL^{-1} with 95% CI.

	KZ52	
	**IC_{50} (µg.mL^{-1})**	**95% CI**
**EBOV-C15**	3.86	2.82 to 4.89
**C15+ WFA**	5.85	4.09 to 7.54
**EBOV-C15-ΔMLD**	2.07	1.86 to 2.28
**EBOV-C15-ΔMLD + WFA**	16.76	13.96 to 19.65

```
                                              4
      GP1                                     0
EBOV  MG-VTGILQLPRDRFKRTSFFLWVIILFQRTFSIPLGVIHNSTLQVSDVDKLVCRDKLSSTNQLRSVGLNLEGNGVATDV
SUDV  ME-GLSLLQLPRDKFRKSSFFVWVIILFQKAFSMPLGVVTNSTLEVTEIDQLVCKDHLASTDQLKSVGLNLEGSGVSTDI
RESTV MGSGYQLLQLPRERFRKTSFLVWVIILFQRAISMPLGIVTNSTLKATEIDQLVCRDKLSSTSQLKSVGLNLEGNGIATDV


EBOV  PSATKRWGFRSGVPPKVVNYEAGEWAENCYNLEIKKPDGSECLPAAPDGIRGFPRCRYVHKVSGTGPCAGDFAFHKEGAF
SUDV  PSATKRWGFRSGVPPKVVSYEAGEWAENCYNLEIKKPDGSECLPPPPDGVRGFPRCRYVHKAQGTGPCPGDYAFHKDGAF
RESTV PSATKRWGFRSGVPPKVVSYEAGEWAENCYNLEIKKSDGSECLPLPPDGVRGFPRCRYVHKVQGTGPCPGDLAFHKNGAF

                                                  2                       2         2
                                                  0                       2         3
                                                  4                       8         8
EBOV  FLYDRLASTVIYRGTTFAEGVVAFLILPQAKKDFFSSHPLREPVNATEDPSSGYYSTTIRYQATGFGTNETEYLFEVDNL
SUDV  FLYDRLASTVIYRGVNFAEGVIAFLILAKPKETFLQSPPIREAVNYTENTSSYYATSYLEYEIENFGAQHSTTLFKINNN
RESTV FLYDRLASTVIYRGTTFAEGVVAFLILSEPKKHFWKATPAHEPVNTTDDSTSYYMTLTLSYEMSNFGGNESNTLFKVDNH

                       2          2                           2
                       5          7                           9
                       9          0                           8        MLD
EBOV  TYVQLESRFTPQFLLQLNETIYTSGKRSNTTGKLIWKVNPEIDTTIGEWAFWETKKNLTRKIRSEELSFTVVSNGAKNIS
SUDV  TFVLLDRPHTPQFLFQLNDTIHLHQQLSNTTGKLIWTLDANINADIGEWAFWENKKNLSEQLRGEELSFETLSLNETEDD
RESTV TYVQLDRPHTPQFLVQLNETLRRNNRLSNSTGRLTWTLDPKIEPDVGEWAFWETKKNFSQQLHGENLHFQILSTHTNNSS


EBOV  GQSPARTSSDPGTNTTTEDHKIMASENSSAMVQVHSQGREAAVS--HLTTLATIS-TSPQSLTTKPGPDNSTHNTPVYKL
SUDV  DATSSRTTKGRISDRATRKYSDLVPKDSPGMVSLHVPEGETTLPSQNSTEGRRVDVNTQETITETTATIIGTNGNNMQIS
RESTV DQSPAGTVQGKISYHPPANNSELVPTDSPPVVSVLTAGRTEEMSTQGLTNGETITGFTANPMTTTIAP------SPTMTS


EBOV  DISEATQVEQHHRRTDNDSTASDTPSATTAAG---PPKAENTNTSKSTDFLDPATTT-SPQNHSETAGNNNTHHQDTGEE
SUDV  TIGTGLSSSQILSSSPTMAPSPETQTSTTYTPKL-PVMTTEESTTPP--RNSPGSTTEAPTLTTPENITTAVKTVLPQES
RESTV EVDNNVPSEQPNNTASIEDSPPSASNETIYHSEMDPIQGSNNSAQSPQTKTTPAPTT-SPMTQDPQETANSSKPGTSPGS

                             Furin cleavage
                                GP1GP2
EBOV  SASSGKLGLITNTIAGVAGLITGGRRTRREAIVNAQPKCNPNLHYWTTQDEGAAIGLAWIPYFGPAAEGIYIEGLMHNQD
SUDV  TSN----GLITSTVTGILGSLGLRKRSRRQVNTRATGKCNPNLHYWTAQEQHNAAGIAWIPYFGPGAEGIYTEGLMHNQN
RESTV AAGPSQPGLTINTVSKVADSLSPTRKQRRSVRQNTANKCNPDLYYWTAVDEGAAVGLAWIPYFGPAAEGIYIEGVMHNQN

                5                                                      6
                6                                                      1
                3                                                      8
EBOV  GLICGLRQLANETTQALQLFLRATTELRTFSILNRKAIDFLLQRWGGTCHILGPDCCIEPHDWTKNITDKIDQIIHDFVD
SUDV  ALVCGLRQLANETTQALQLFLRATTELRTYTILNRKAIDFLLRRWGGTCRILGPDCCIEPHDWTKNITDKINQIIHDFID
RESTV GLICGLRQLANETTQALQLFLRATTELRTYSLLNRKAIDFLLQRWGGTCRILGPSCCIEPHDWTKNITDEINQIKHDFID

EBOV  KTLPDQGDNDNWWTGWRQWIPAGIGVTGVIIAVIALFCICKFVF
SUDV  NPLPNQDNDDNWWTGWRQWIPAGIGITGIIIAIIALLCVCKLLC
RESTV NPLPDHGDDLNLWTGWRQWIPAGIGIIGVIIAIIALLCICKILC
```

Supplementary Figure 1. Alignment of *Zaire ebolavirus* (EBOV), *Sudan ebolavirus* (SUDV) and *Reston ebolavirus* (RESTV) $GP_{1,2}$ amino acid sequences. The signal peptide is indicated in red text. The mature GP_1 is highlighted in green, with the mucin-like domain indicated in brown. GP_2 is highlighted in blue. N-linked glycosylation sequons are highlighted in yellow, with sites in GP_1 and GP_2 indicated with the numbering from the EBOV genome sequence (Uniprot accession number Q05320).

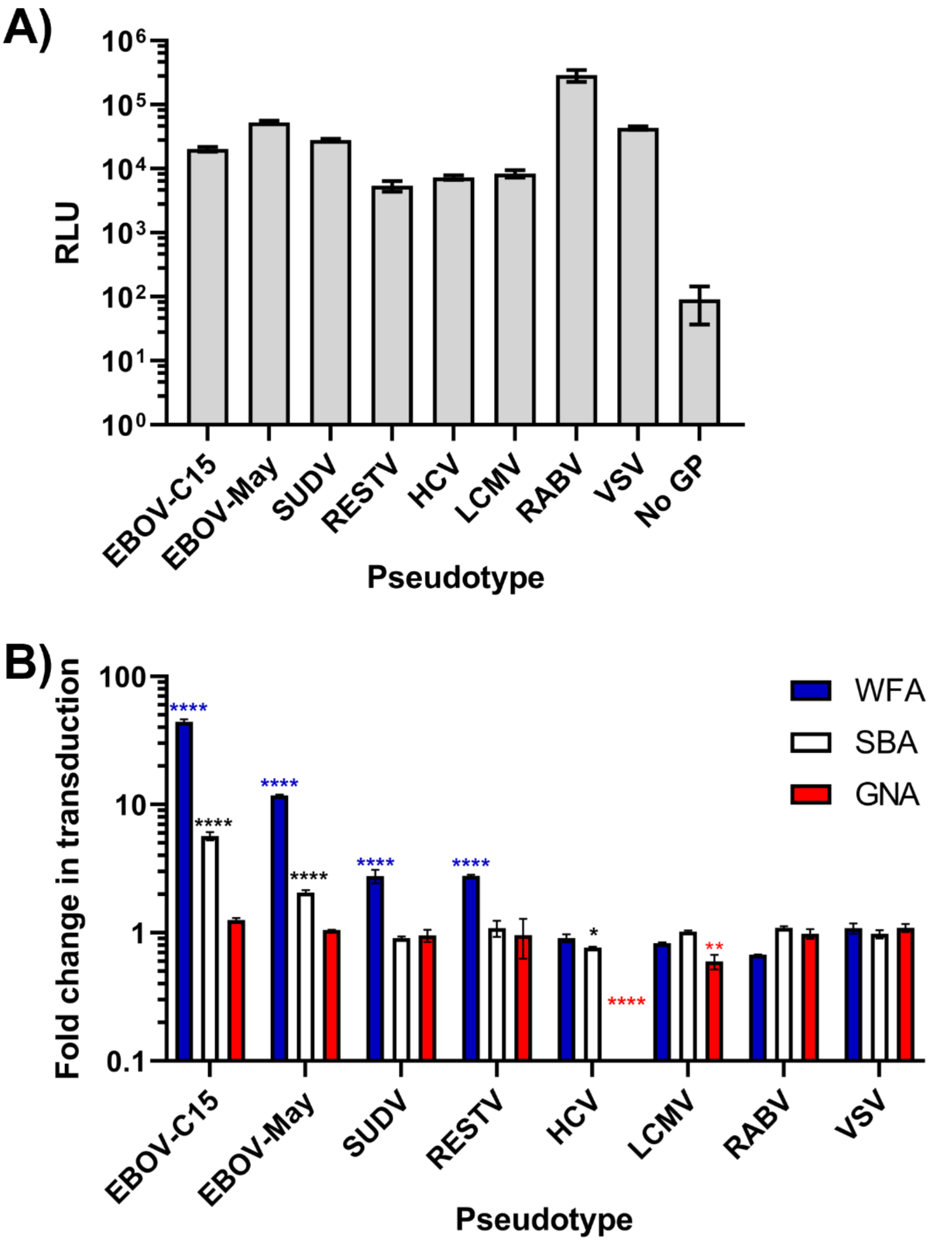

Supplementary Figure 2. A) Luciferase activity of HuH7 cells transduced by the panel of PVs. **B)** Comparison between the mean fold change in transduction of each PV with lectin at a concentration of 20 µg.mL^{-1} and a no lectin control. Statistical significance was determined by one-way ANOVA followed by Dunnett's multiple comparison test, p<0.05 (*), p<0.01 (**), p>0.0001 (****). Comparisons with p>0.05 are not labelled. Data from a single representative experiment. Each data point represents the mean of three technical repeats with errors displaying SD.

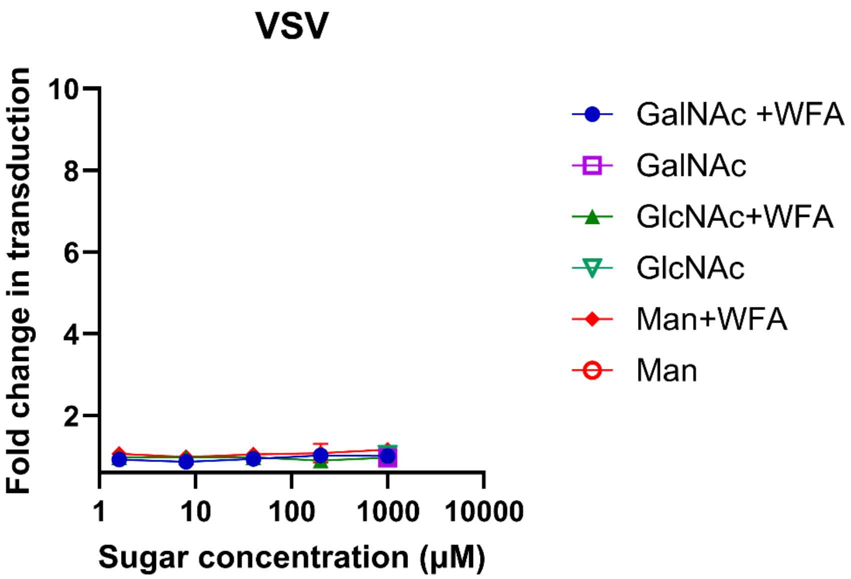

Supplementary Figure 3. Effect of sugars (GalNAc, GlcNAc, mannose (Man)) on transduction of HuH7 cells with VSV pseudotypes. WFA was used at a concentration of 10µg.mL^{-1}. Statistical significance was determined by one-way ANOVA on samples treated with the greatest concentration of each sugar, followed by Dunnett's multiple comparison test. Data from a single representative experiment. Each data point represents the mean of three technical repeats with errors displaying SD.

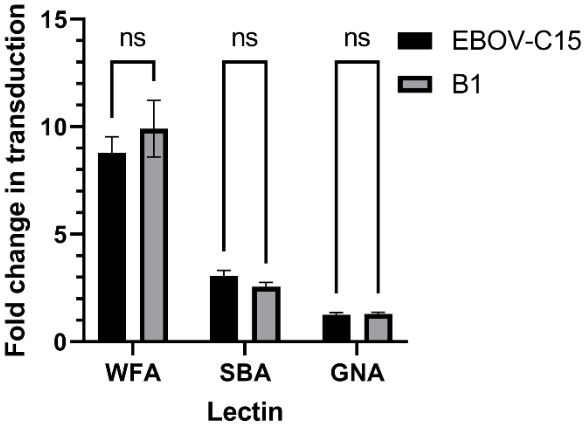

Supplementary Figure 4. Enhancement of transduction of HuH7 cells with *Ebolavirus* GP variants EBOV-C15 and EBOV B1 with WFA, SBA or GNA used at a concentration of 10µg.mL^{-1}. Statistical significance was determined by one-way ANOVA followed by Sidak's multiple comparison, $p>0.05$ (ns). Data from a single representative experiment. Each data point represents the mean of three technical repeats with errors displaying SD.

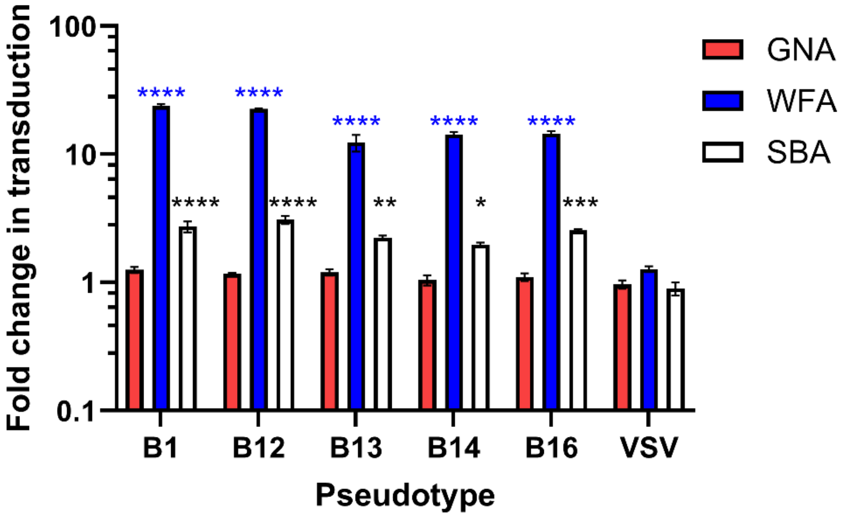

Supplementary Figure 5. Enhancement of Ebola virus variants EBOV B1, B12, B13, B14, B16 with WFA, SBA or GNA used at a concentration of 10 µg.mL^{-1}. Pseudotypes possessing the VSV glycoprotein were used as a control in this experiment. Statistical significance was determined by one-way ANOVA followed by Dunnett's multiple comparison test, $p<0.05$ (*), $p<0.01$ (**), $p>0.0001$ (****). Comparisons with $p>0.05$ are not labelled. Data from a single representative experiment. Each data point represents the mean of three technical repeats with errors displaying SD.

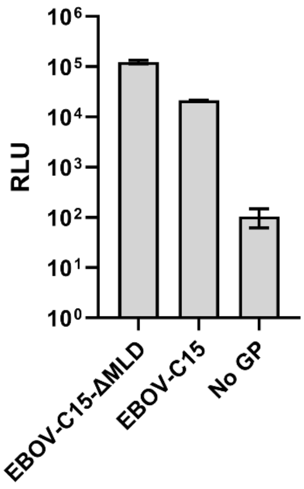

Supplementary figure 6. Transduction of HuH7 cells by pseudotypes bearing the EBOV-C15 and EBOV-C15-ΔMLD glycoproteins. Transduction was measured in relative light units (RLU) as a measure of luciferase reporter expression. Data from a single representative experiment. Each data point represents the mean of three technical repeats with errors displaying SD.
